# Excitation Intensity-Dependent Quantum Yield of Semiconductor Nanocrystals

**DOI:** 10.1021/acs.jpclett.3c00143

**Published:** 2023-03-09

**Authors:** Subhabrata Ghosh, Ulrich Ross, Anna M. Chizhik, Yung Kuo, Byeong Guk Jeong, Wan Ki Bae, Kyoungwon Park, Jack Li, Dan Oron, Shimon Weiss, Jörg Enderlein, Alexey I. Chizhik

**Affiliations:** a Third Institute of Physics − Biophysics, Georg August University Göttingen, Friedrich-Hund Platz 1, 37077 Göttingen, Germany; b IV. Physical Institute - Solids and Nanostructures, Georg August University Göttingen, Friedrich-Hund Platz 1, 37077 Göttingen, Germany; c Department of Chemistry and Biochemistry, University of California Los Angeles, Los Angeles, California 90095, United States; d School of Chemical and Biomolecular Engineering, Pusan National University, Busan 46241, Republic of Korea; e SKKU Advanced Institute of Nanotechnology (SAINT), Sungkyunkwan University, Suwon 16419, Republic of Korea; f Korea Electronics Technology Institute, Seongnam-si, Gyeonggi-do 13509, Republic of Korea; g Department of Molecular Chemistry and Materials Science, Weizmann Institute of Science, Rehovot 76100, Israel; h California NanoSystems Institute, University of California Los Angeles, Los Angeles, California 90095, United States; i Department of Physiology, University of California Los Angeles, Los Angeles, California 90095, United States; j Department of Physics, Institute for Nanotechnology and Advanced Materials, Bar-Ilan University, Ramat-Gan, 52900, Israel; k Cluster of Excellence “Multiscale Bioimaging: from Molecular Machines to Networks of Excitable Cells,” (MBExC), Georg August University of Göttingen, Robert-Koch-Str. 40, 37075 Göttingen, Germany

## Abstract

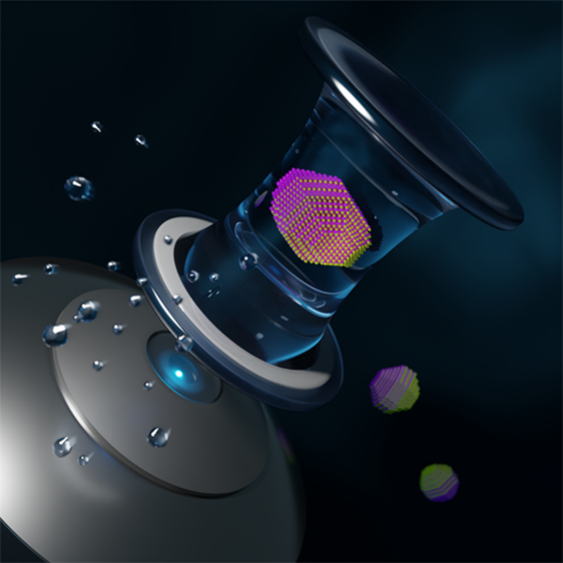

One of the key phenomena that determine the fluorescence of nanocrystals is the nonradiative Auger-Meitner recombination of excitons. This nonradiative rate affects the nanocrystals’ fluorescence intensity, excited state lifetime, and quantum yield. Whereas most of the above properties can be directly measured, the quantum yield is the most difficult to assess. Here we place semiconductor nanocrystals inside a tunable plasmonic nanocavity with subwavelength spacing and modulate their radiative de-excitation rate by changing the cavity size. This allows us to determine absolute values of their fluorescence quantum yield under specific excitation conditions. Moreover, as expected considering the enhanced Auger-Meitner rate for higher multiple excited states, increasing the excitation rate reduces the quantum yield of the nanocrystals.

The discovery of luminescent semiconductor nanocrystals has become one of the major events in the modern era of nanotechnology and photonics.^1−6^ Despite numerous studies of their optical properties and significant advances in their synthesis that have been achieved in recent years, semiconductor nanocrystals still show a number of drawbacks that limit their application in many fields.^7^ One of the major problems for their use in fluorescence imaging and spectroscopy is nonradiative Auger-Meitner recombination.^8−10^ Its rate depends on a number of intrinsic parameters, such as nanoparticle size, core/shell structure, or lattice defects.^11^ This recombination limits the efficiency of nanocrystal photon emission when they are subjected to saturating excitation, corresponding to the formation of multiple excited states. As a result, increase of the excitation intensity leads to the sublinear growth of the nanocrystals’ brightness and decrease of their average excited state lifetime.^12−14^

The photon emission efficiency of nanocrystals is quantified by the fluorescence quantum yield which is typically measured using either a comparative method (referencing against a sample of known quantum yield) or employing an integrating sphere.^15^ These methods allow one to determine an accurate value for ensembles of nanocrystals in solution. However, since the excitation light is focused within a relatively large volume, both of them do not allow one to precisely control the excitation field density.^16^ Therefore, obtained quantum yield values correspond to an undefined probability of Auger-Meitner recombination. Bawendi and co-workers have determined the quantum yield of the biexciton state in semiconductor nanocrystals using antibunching measurements.^17^ Subsequently, the quantum yield of biexcitons has been the subject of many recent studies.^18−25^

Here, we determine the excitation intensity-dependent quantum yield of semiconductor nanocrystals using a fundamentally different approach that is based on modulating their radiative transition rate with a plasmonic nanocavity.^26−30^ Placing a semiconductor nanocrystal inside a nanocavity changes the local density of state (LDOS) of the electromagnetic field around the particle and thereby modulates the probability of its radiative de-excitation rate.^28,31,32^ Modulation of the radiative rate has been demonstrated for immobilized fluorophores placed close to a dielectric interface,^33,34^ a sharp tip of a scanning probe microscope,^35^ a metallic mirror,^36,37^ or diverse combinations of metallic nanoparticles.^38−41^ Placing a fluorophore inside a planar nanocavity allows for precise and easy monitoring of LDOS by measuring the distance between the cavity mirrors. The theoretical model that we developed takes into account all the aspects of the radiative rate modulation by the nanocavity for the specific optical properties of semiconductor nanocrystals.^27^ By comparing the modeled radiative rate modulation with the actually measured one, we deduce the nanocrystals’ quantum yield. For the details of this nanocavity-based method of quantum yield determination, see ref (27). Focusing excitation light within a diffraction-limited focal spot and using time-correlated single photon counting (TCSPC) fluorescence detection allowed us to derive the relation between the excitation field density and the quantum yield. Since the LDOS within the cavity changes for both neutral and charged states of the nanoparticle, the method allows us to measure the total average quantum yield of the nanocrystal at any excitation intensity. This method indirectly provides a relatively easy access to the quantum yield of highly excited states, where measurement of photon statistics becomes difficult due to the scarcity of the correlation signal.

We studied red-emitting CdS/CdSe/CdS spherical nanocrystals that have *quasi*-type-II band alignment in which an excited electron is delocalized over the entire nanocrystal while the hole remains localized in the CdSe emissive layer (see Figure 1a). Ensemble absorption and emission spectra of particles dispersed in toluene are shown in Figure 1b. Nanocrystals synthesis followed the protocol reported in ref (42) and is described in the SI. Such nanocrystals not only can be used for common applications as a replacement for standard type-I particles, but also are promising candidates for local voltage sensing and optical gain applications.^43^ High-resolution transmission electron microscopy (HRTEM) images of nanocrystals are shown in Figure 1b. They confirm that the nanocrystals have a spherical shape with diameters of 16 ± 3 nm (see also Figure S1 of the SI for more images). In the HRTEM images, no clear distinction between CdS core, CdSe emissive layer, and CdS is visible due to lattice similarities of CdS core and CdS outer shell.^44^

**Figure 1:**
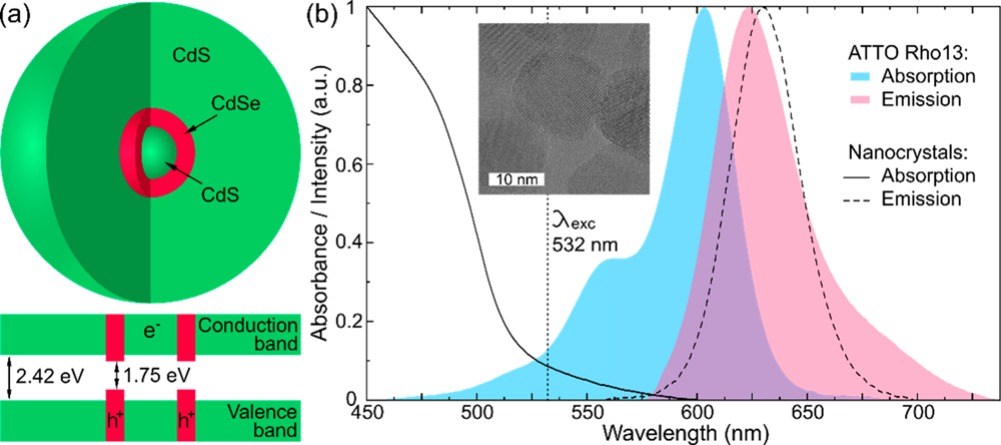
(a) Schematic of the CdS/CdSe/CdS core–shell–shell nanocrystals and its conduction and valence band structure. The band gaps for CdS and CdSe are given for bulk material. (b) Absorption and emission spectra of CdS/CdSe/CdS nanocrystals dispersed in toluene and of ATTO Rho13 dye in aqueous solution. Inset shows high resolution transmission electron microscopy image of CdS/CdSe/CdS nanocrystals.

Increase of excitation light intensity leads to a decrease of the average excited state lifetime of the nanocrystals (red curve in Figure 2a) caused by the trapping of excessive charges that are generated inside the nanocrystal and as a result, to an increase of the probability for nonradiative Auger-Meitner recombination. The decreased lifetime is also accompanied by a saturation in fluorescence intensity (blue curve in Figure 2a) and presumably quantum yield of the emitters. Fluorescence decay curves measured at the highest and lowest excitation intensity (Figure 2 (b) and (c), respectively) demonstrate a substantial increase of the de-excitation rate upon increase of the excitation intensity. Since fluorescence decay curves of semiconductor nanocrystals typically consist of multiple monoexponential components, our fitting model used up to 100 monoexponential functions that allowed us to precisely determine the average excited state lifetimes independent of the complexity of the curve.

**Figure 2:**
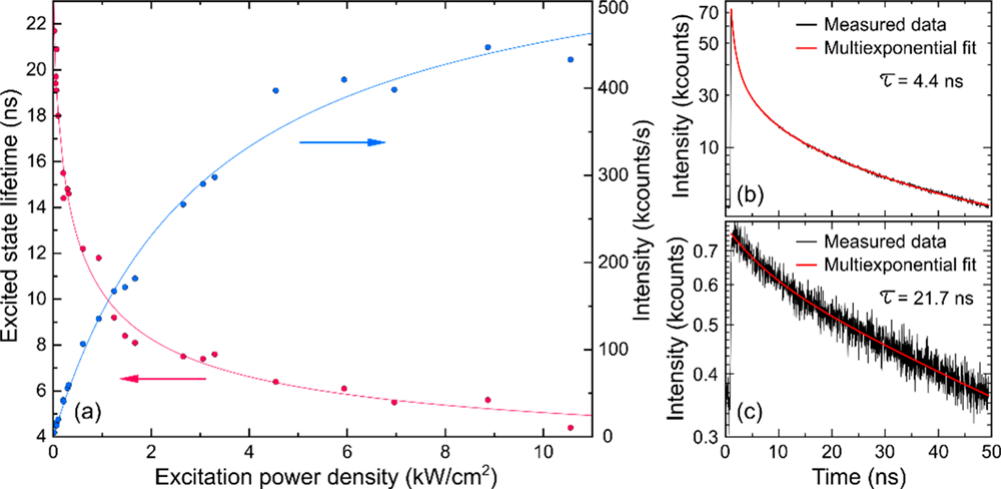
(a) Dependence of the excited state lifetime (red solid circles) and emission intensity (blue solid circles) of CdS/CdSe/CdS nanocrystals on excitation intensity. The red and blue curves are the fits with sigmoid and saturation function, respectively. Black curves in (b) and (c) are fluorescence decay curves measured at highest and lowest excitation intensity, respectively. Red curves are multiexponential tail fits; τ is the average excited state lifetime.

Whereas fluorescence lifetime and intensity can be directly measured using TCSPC, the quantum yield is typically determined in a comparative manner against a reference sample. In contrast to a confocal microscope, where excitation light is focused within a diffraction-limited focal spot, conventional spectrometers that are used for referential quantum yield measurements are usually done at fixed (and arbitrary) intensity. As a result, quantum yield measurements of semiconductor nanocrystals under strong excitation using a reference sample are prone to errors due to an undefined nonradiative Auger-Meitner recombination rate. Single particle nanocavity-based measurements could also potentially allow one to deduce the quantum yield of bright and gray states of nanocrystals. This, however, would require significantly more complex measurements, including axial localization of the nanoparticle between the mirrors and three-dimensional orientation of its emission transition dipole.

In this study, we examine the difference between the results of quantum yield measurements using a comparative method and an absolute nanocavity-based technique. Nanocavity-based QY measurements were performed using a confocal microscope that was equipped with a pulsed excitation laser and a single photon avalanche diode with time-correlated single photon counting electronics for lifetime measurements (Figure 3). The nanocavity consists of two silver layers that were deposited by vapor deposition onto the surfaces of a glass cover slide (30 nm, bottom mirror) and a plano-convex lens (60 nm, upper mirror), respectively (inset (a) of Figure 3). Prior to silver deposition, glass substrates were coated with a 2 nm thick titanium layer for better adhesion of silver. The thicker upper mirror maximized the collection efficiency of the fluorescence that was mostly transmitted through the bottom mirror toward the high numerical aperture objective lens. The spherical shape of the upper mirror allows us to modulate the distance between the mirrors by laterally moving the cavity with respect to the excitation focus with a piezo scan stage. For a given lateral laser focus position, the exact distance between the cavity mirrors was determined by measuring a white light transmission spectrum using a broad-band halogen lamp. For the excited state lifetime measurements, a droplet of toluene solution of nanocrystals was placed between the cavity mirrors. The lifetime measurements were done across the first interference ring (λ/2 region) of the cavity that can be seen in the white light transmission pattern as the first color ring around the center of the cavity (inset (b) in Figure 3), a region where the lifetime modulation of a fluorophore is maximized. All the measurements were done using the 532 nm light of a supercontinuum laser. Further details of the experimental setup can be found in the SI.

**Figure 3:**
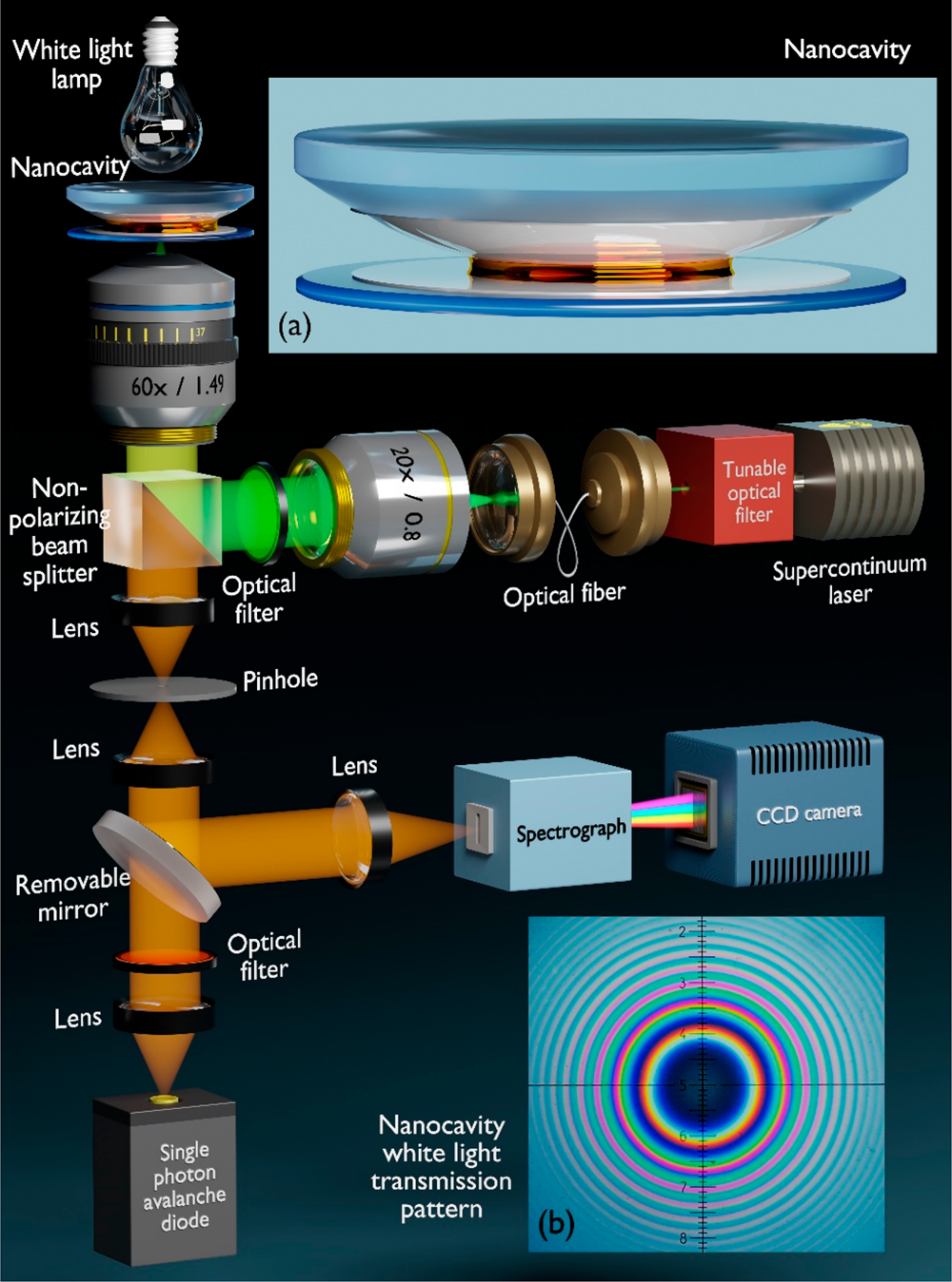
Schematic of the custom-built confocal microscope used for quantum yield measurements. Inset (a) shows a schematic of the nanocavity with a droplet of toluene solution with semiconductor nanocrystals between the mirrors. Inset (b) shows the white light transmission pattern around the center of the cavity (Newton rings). The first colored ring corresponds to the λ/2 region of the cavity.

We performed cavity-modulated lifetime measurements of nanocrystals at four different excitation intensities between 0.07 and 10.6 kW/cm^2^, see Figure 1a. Open circles in Figure 4 show results for the excited state lifetime measurements of particles at different cavity lengths. For each data point, both the fluorescence decay and the white light transmission spectrum were measured. The obtained curve was fitted with a theoretical model that takes into account all the parameters of the optical system and the electrodynamic coupling of a quantum emitter to a planar metallic nanocavity. The model explicitly takes into account the orientation-dependent interaction of nanocrystals with the cavity, assuming an isotropic excitation and emission dipole structure of the nanocrystals.^26^ The only free parameters of this model are the quantum yield and the free-space (out-of-cavity) lifetime values. A complete description of the model can be found in ref (27). Comparison of the free-space lifetime as calculated from the nanocavity measurements with a value measured in a droplet of solution placed on a clean glass cover slide allows us to estimate the reliability of the obtained quantum yield value.

**Figure 4:**
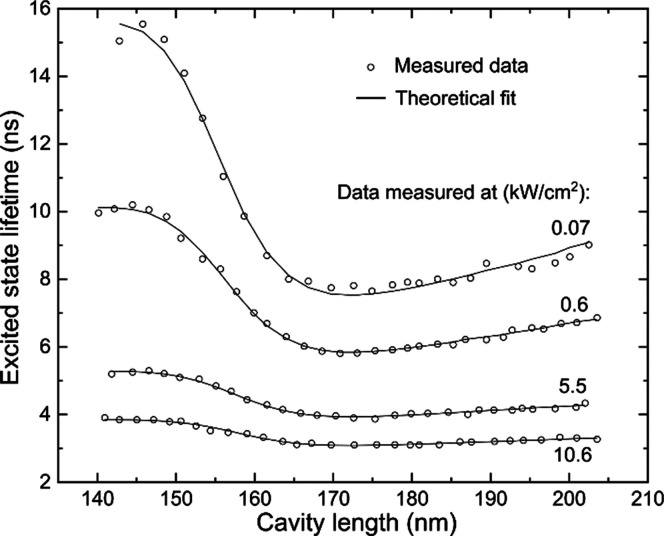
Average excited state lifetime of semiconductor nanocrystals as a function of the cavity length measured at four different excitation intensities. Circles are measured data; solid lines are theoretical fits. Fit results are given in Table 1.

The theoretical fits (solid curves in Figure 4) show that tuning the excitation intensity drastically changes the cavity-modulated average excited state lifetime of nanocrystals. In particular, both absolute lifetime values and the amplitude of the nanocavity-induced lifetime modulation are maximal at lowest excitation intensity. Now we would like to discuss how exactly the modulation of the excitation intensity changes the cavity-modulated lifetime of nanoparticles. Placing an emitter between the cavity mirrors alters the electromagnetic field LDOS around it, which changes the *radiative* rate of the emitter. The *nonradiative* recombination rate of the particles is determined by the interaction of excitons with excessive charges and is not affected by LDOS. Increasing the excitation intensity enhances the probability of the generation of excessive charges inside the nanocrystals. This, in turn, increases the nonradiative Auger-Meitner rate and, hence, increases the total de-excitation rate and shortens the average excited state lifetime of the nanocrystal. Since the nonradiative rate becomes dominant at high excitation intensity, the nanocavity-induced modulation of the radiative rate results in a *smaller modulation amplitude* of the total de-excitation rate. Since the latter can be directly measured as the average excited state lifetime of the nanocrystals, we observe lower amplitude to the cavity-induced lifetime modulation at higher excitation intensity.

Table 1 shows the measured quantum yield and lifetime values of the nanoparticles. The difference between the measured and calculated free space lifetime values does not exceed a few percent that assures the reliability of calculated quantum yield values. The obtained quantum yield values show an inverse correlation with the excitation intensity: The highest quantum yield is obtained for the lowest excitation intensity used, while an increase of excitation intensity leads to a reduction of both quantum yield and lifetime. The value obtained at lowest excitation intensity is in good agreement with previously reported values of near-unity quantum yield for semiconductor nanocrystals when the average number of excitons created per pulse per particle is significantly lower than one.^42,45,46^

**Table 1:** Results of Nanocavity-Based Quantum Yield and Lifetime Measurements of Semiconductor Nanocrystals Using Different Excitation Intensities^a^

Exc. intensity (kW/cm^2^)	Lifetime measured (ns)	Lifetime calculated (ns)	Quantum yield (%)
0.07	20.9 ± 0.05	19.5 ± 0.1	91 ± 3
0.6	12.2 ± 0.05	11.9 ± 0.1	59 ± 2
5.5	6.1 ± 0.05	5.8 ± 0.1	26 ± 2
10.6	4.4 ± 0.05	4.2 ± 0.1	19 ± 2
Referential			42 ± 3

a The selected excitation intensity values correspond to certain integer values of the total excitation intensity of the laser measured before the objective lens of the microscope. The row “Referential” shows the results of the quantum yield measurement by referencing against ATTO Pho13 dye.

For the comparative measurements, we reference the nanocrystal emission against the emission of the organic dye ATTO Rho13 that emits in the same spectral range as the nanocrystals (Figure 1). Whereas absorption spectra of the dye and nanocrystals have significantly different shapes, their partial overlap in the green spectral range allowed us to use the same excitation wavelength of 532 nm where both emitters have similar excitation efficiencies. To determine a quantum yield of the nanocrystals referenced against that of the dye, fluorescence intensities of reference dye and nanocrystals were measured over a range of fluorophore concentrations corresponding to five different extinction values at the excitation wavelength. This allowed us to minimize inaccuracies of the quantum yield measurement due to errors in the optical absorption measurements. Sample concentrations were chosen in such a way that the maximum extinction did not exceed 0.1, so that nonlinear reabsorption processes can be neglected. The details of the referential quantum yield measurements are provided in the SI. To verify the literature value of ATTO Rho13, we also measured the absolute quantum yield of the dye using the nanocavity-based method. The obtained value is 82%. The results of the nanocavity-based quantum yield measurements of the dye and the comparative measurements of nanocrystals are shown in Table 1. The quantum yield of 42% that was obtained using the comparative method suggests that the used excitation intensity leads to multiexciton generation which enhances the nonradiative de-excitation rate and thus reduces the average quantum yield of the nanocrystals. At the same time, comparative quantum yield measurements do not allow one to monitor the probability of multiexciton generation neither by monitoring the shape of the fluorescence decay curve, nor by antibunching measurements.

The nonlinear dependence of quantum yield, lifetime, and emission intensity of the particles on excitation intensity demonstrates the highly complex mechanisms of single and multiexciton generation in semiconductor nanocrystals. Therefore, their quantum yield measurements require not only the study of their photophysical properties, but also a precise control of all the experimental parameters, which is not always possible with conventional methods. The above results show that the nanocavity-based method allows for an easy sample preparation and assembly, accurate control of the distance between the mirrors, and precise modeling of the fluorophores radiative rate modulation. The method can be applied not only to various complex systems where conventional methods fail,^28,29,47,48^ but can be used at the single molecule level.^49^ As the excitation intensity-tuned lifetime has been recently used for enhancing spatial resolution in fluorescence imaging,^50^ the nanocavity-based method could potentially help to measure single particle quantum yield directly within the biological sample.

In summary, by placing semiconductor nanocrystals inside a plasmonic nanocavity we tailored their radiative rate to deduce the absolute quantum yield. We showed that the modulation of the excitation power density changes the probability of Auger-Meitner recombination and, hence, the quantum yield of nanoparticles by a factor of almost five. We envision that the nanocavity-based measurement of quantum yield will allow for further understanding of a complex relation between optical properties of semiconductor nanocrystals.

## Supporting Information Available

Additional experimental details, methods and materials, including TEM images of nanocrystals (PDF)
