# Supplementary material for: Excitation Intensity-Dependent Quantum Yield of Semiconductor Nanocrystals
Source: J Phys Chem Lett. 2023 Mar 9;14(10):2702–7. doi: 10.1021/acs.jpclett.3c00143 (PMC10026174; doi:10.1021/acs.jpclett.3c00143)
Supplement: Supplementary file 1 — jz3c00143_si_001.pdf [file jz3c00143_si_001.pdf]

Supporting Information

Excitation Intensity-Dependent Quantum Yield of Semiconductor Nanocrystals

Subhabrata Ghosh,[1] Ulrich Ross,[2] Anna M. Chizhik,[1] Yung Kuo,[3] Byeong Guk Jeong,[4] Wan Ki Bae,[5] Kyoungwon Park,[6] Jack Li,[3] Dan Oron,[7] Shimon Weiss,[3,8,9,10] Jörg Enderlein,[1,11] Alexey I. Chizhik[1]

[1]Third Institute of Physics – Biophysics, Georg August University Göttingen, Friedrich-Hund Platz 1, 37077 Göttingen, Germany
[2]IV. Physical Institute - Solids and Nanostructures, Georg August University Göttingen, Friedrich-Hund Platz 1, 37077 Göttingen, Germany
[3]Department of Chemistry and Biochemistry, University of California Los Angeles, Los Angeles, CA 90095, United States
[4]School of Chemical and Biomolecular Engineering, Pusan National University, Busan 46241, Republic of Korea
[5]SKKU Advanced Institute of Nanotechnology (SAINT), Sungkyunkwan University, Suwon 16419, Republic of Korea
[6]Korea Electronics Technology Institute, Seongnam-si, Gyeonggi-do 13509, Republic of Korea
[7]Department of Molecular Chemistry and Materials Science, Weizmann Institute of Science, Rehovot 76100, Israel
[8]California NanoSystems Institute, University of California Los Angeles, Los Angeles, CA 90095, United States
[9]Department of Physiology, University of California Los Angeles, Los Angeles, CA 90095, United States
[10]Department of Physics, Institute for Nanotechnology and Advanced Materials, Bar-Ilan University, Ramat-Gan, 52900, Israel
[11]Cluster of Excellence "Multiscale Bioimaging: from Molecular Machines to Networks of Excitable Cells," (MBExC), Georg August University of Göttingen, Robert-Koch-Str. 40, 37075 Göttingen, Germany

Materials

CdO (99.95%), oleic acid (OA, 99%), tri-n-octyl phosphine (TOP, 99%), selenium (Se, 99.99%, powder), and sulfur (S, 99.998%, powder) were purchased from UniAm. 1-Dodecanethiol (DDT, ≥98%), and 1-octadecene (ODE, 90%, technical grade) were purchased from Sigma-Aldrich. Organic solvents were purchased from Samchun chemicals. All chemicals were used as received. Synthetic procedures were conducted under an inert atmosphere with the Schlenk line.

Preparation of stock solutions

Preparation of stock solutions [0.5 M cadmium oleate ($Cd(OA)_2$) in ODE (100 mmol), 2 M selenium in TOP (TOPSe, 20 mmol), mixed Cd-Se solution (0.1 M $Cd(OA)_2$ and TOPSe in ODE), 0.25 M sulfur dissolved in ODE (S-ODE) (10 mmol), and 0.5 M DDT in ODE (100 mmol)] was conducted prior to synthetic procedures. All stock solutions were stored in a glovebox.

Synthesis of CdS/CdSe/CdS nanocrystals

Synthetic procedures of CdS/CdSe/CdS nanocrystals were followed by a previous report [1]. Synthesis of CdS seeds was first conducted in a three-neck flask. 0.6 mL of $Cd(OA)_2$ stock solution and 9 mL of ODE were loaded in the flask. The flask was heated to 270 °C, and then 0.5 mL of S-ODE was quickly injected into the flask. The flask cooled to 250 oC for the growth of CdS seeds. After 10 mins, the flask cooled down to room temperature to complete the reaction. The resultant nanocrystals were mixed with an excess amount of ethanol and re-dispersed in toluene. To grow a CdSe emissive layer, 100 mg of CdS seeds and 10 mL of ODE were mixed in a three-neck flask and put under vacuum at 110 oC for 1 hr. After filling with Ar, the flask heated to 300 oC. At the reaction temperature, the desired amount of mixed Cd-Se stock solutions was injected into the flask at a constant rate of 5 mL/hr. The flask cooled to room temperature after the injection. The purification was repeated with ethanol/toluene and re-dispersed in toluene. For further growth of a CdS Shell, 100 mg CdS/CdSe NCs and 10 mL of ODE were loaded into a three-neck flask. The flask was degassed at 110 oC and heated to 300 oC. An alternate injection of $Cd(OA)_2$ and the DDT stock solution proceeded into the flask at a rate of 2 mL/hr. After 1hr of reaction, the flask was cooled to room temperature. The crude NC solution was purified by the precipitation (ethanol)/dispersion (toluene) method. The resulting NCs were re-dispersed in toluene for further use.

Confocal microscope

Fluorescence lifetime measurements were performed with a custom-built confocal microscope equipped with an objective lens of high numerical aperture (Apo N, 60×/1.49 NA oil immersion, Olympus). A white light laser system (Fianium SC400-4-20) with a tunable filter (AOTFnC-400.650-TN) served as excitation source. Excitation wavelength for all the single particle measurements was 485 nm. Collected fluorescence was focused onto the active area of a single photon detection module (MPD series, PDM). Data acquisition was accomplished with a multichannel picosecond event timer (PicoQuant HydraHarp 400). Photon arrival times were histogrammed (bin width of 32 ps) for obtaining fluorescence decay curves. Fluorescence spectra were measured using an Andor SR 303i spectrograph and a CCD camera (Andor iXon DU897 BV).

TEM imaging

TEM imaging was carried out at 300 keV on a FEI Titan G2 ETEM, equipped with a CEOS image corrector. Nanoparticles were dispersed in the solution via ultrasound and subsequently drop-cast onto standard amorphous carbon membranes (Plano).

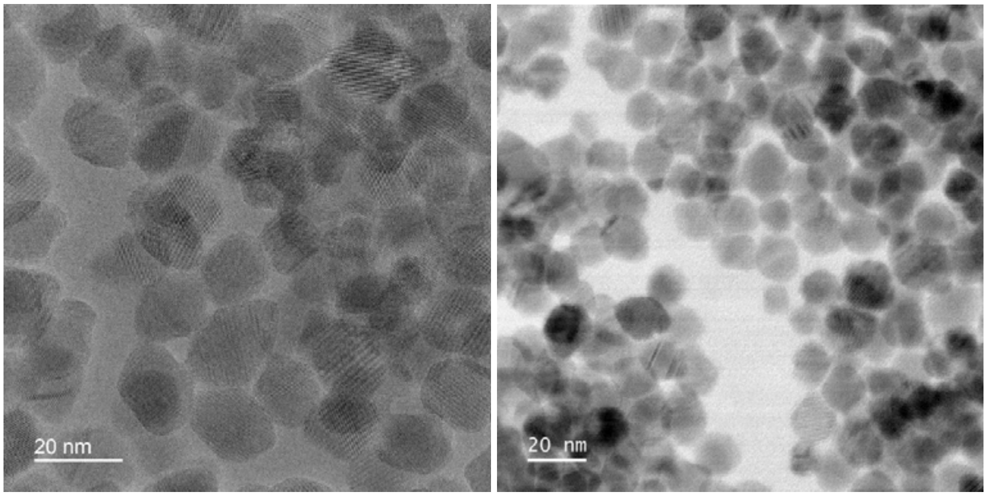

Figure S1: HR-TEM image (left) and BF-STEM image (right) of CdS/CdSe/CdS spherical quantum well nanocrystals showing slight variation of size and shapes of particles.

Quantum yield measurement using the comparative method

A standard 10 mm cuvette was used to measured quantum yield by reference sample method. The concentrations of the samples were chosen in such way that maximum value of absorbance not exceeded 0.1 at excitation wavelength and above, to reduce the re-absorption and other non-linear effect which perturbed the quantum yield values. The measurement protocols are as follows:

(1) Recording of absorption spectra at different concentrations of the sample not exceeded the absorbance of 0.1 at excitation wavelength.

(2) Recording of emission spectra from the same concentrations at which absorption was measured.

(3) Plotting the graph of integrated fluorescence intensity vs. absorbance and fitting with a straightline.

(4) Followed the same for reference samples e.g. dye molecules which have chosen in such a way that absorptions and emissions of dye molecules have at similar wavelength with the unknown samples.

(5) To calculate the quantum yield the following formulae was used:

$$\Phi_{unknown\ sample} = \Phi_{reference} \frac{k_{unknown\ sample}}{k_{reference}} \frac{n_{unknown\ sample}}{n_{reference}} \qquad (5),$$

where Φ is quantum yield *k* is the gradient obtained from integrated fluorescence intensity vs. absorbance plot, *n* is refractive index of the solvent. $\Phi_{reference}$ = 82% was obtained using the nanocavity-based method.

References

1. B. G. Jeong et. al., ACS Nano 2016, 10, 10, 9297-9305
